# Strong and Atmospherically Stable Dicationic Oxidative Dopant

**DOI:** 10.1002/advs.202101998

**Published:** 2021-10-28

**Authors:** Tadanori Kurosawa, Toshihiro Okamoto, Yu Yamashita, Shohei Kumagai, Shun Watanabe, Jun Takeya

**Affiliations:** ^1^ Material Innovation Research Center (MIRC) and Department of Advanced Materials Science Graduate School of Frontier Sciences The University of Tokyo 5‐1‐5 Kashiwanoha Kashiwa Chiba 277‐8561 Japan; ^2^ AIST‐UTokyo Operando‐Measurement Technology Open Innovation Laboratory (OPERANDO‐OIL) National Institute of Advanced Industrial Science and Technology (AIST) 5‐1‐5 Kashiwanoha Kashiwa Chiba 277‐8561 Japan; ^3^ PRESTO JST, 4‐1‐8 Honcho Kawaguchi Saitama 332‐0012 Japan; ^4^ International Center for Materials Nanoarchitectonics (WPI‐MANA) National Institute for Materials Science (NIMS) 1‐1 Namiki Tsukuba Ibaraki 205‐0044 Japan

**Keywords:** atmospheric stability, dicationic salt, p‐dopant, strong doping ability

## Abstract

Increasing the doping level of semiconducting polymer using strong dopants is essential for achieving good electrical conductivity. As for p‐dopant, raising the electron affinity of a neutral compound through the dense introduction of electron‐withdrawing group has always been the predominant strategy to achieve strong dopant. However, this simple and intuitive strategy faces extendibility, accessibility, and stability issues for further development. Herein, the use of dicationic state of tetraaryl benzidine (TAB^2+^) in conjunction with bis(trifluoromethylsulfonyl)imide anion (TFSI^−^) as a strong and atmospherically stable p‐dopant (TAB–2TFSI), for which the concept is hinted from a rapid and spontaneous dimerization of radical cation dopant, is demonstrated. TAB–2TFSI possesses a large redox potential such that it would have deteriorated when in contact with H_2_O. However, no trace of degradation after 1 year of storage under atmospheric conditions is observed. When doping the state‐of‐the‐art semiconducting polymer with TAB–2TFSI, a high doping level together with significantly enhanced crystallinity is achieved which led to an electrical conductivity as high as 656 S cm^−1^. The concept of utilizing charged molecule as a dopant is highly versatile and will potentially accelerate the development of a strong yet stable dopant.

## Graphical abstract

The use of charged molecules as a strong and atmospherically stable p‐dopant is demonstrated. Despite showing a strong doping ability above −5.7 eV, the newly reported dopant shows no trace of deterioration upon storage under ambient conditions over a year. A highly ordered cocrystal structure is formed with polythiophene, leading to an electrical conductivity as high as 650 S cm^−1^.

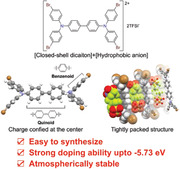

## 1 Introduction

Since the discovery of the synthesis of polyacetylene film and its heavily doped state back in the 1970s,^[^ ^1^, ^2^, ^3^ ^]^ the development of conducting polymers has constantly been one of the mainstream for the realization of organic electronics. Because the electrical conductivity of doped polymer is proportional to the product of charge carrier density and carrier mobility, the most feasible strategy for achieving high conductivity will be heavily doping semiconducting polymer with high carrier mobility. With this in mind, intensive research striving for better conducting polymer systems has been carried out from both dopant and semiconducting polymer viewpoints as well as seeking wider applications.^[^ ^4^, ^5^, ^6^, ^7^ ^]^

**Figure 1:**
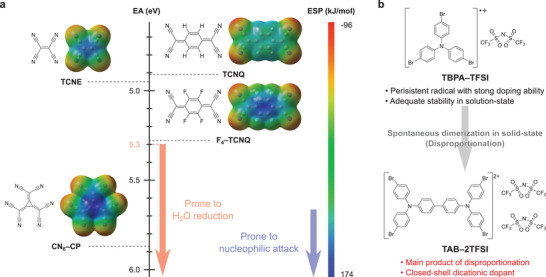
a) EA and electrostatic potential (ESP) map of representative neutral dopants. ESP values were calculated at the B3LYP/6‐31+G (d) level of theory. b) Chemical structures of TBPA–TFSI and TAB–2TFSI in this work.

The development of strong and efficient organic dopant is intuitively achieved by increasing the electron affinity (EA) or decreasing the ionization potential (IP) as much as possible for p‐ or n‐doping, respectively.^[^ ^8^ ^]^ As for p‐dopant, this design strategy is usually translated into extended *π*‐conjugation and (or) dense introduction of electron‐withdrawing groups on a single core represented by the tetracyanoquinodimethane (TCNQ) derivatives.^[^ ^9^, ^10^, ^11^ ^]^ As for the extreme case, hexacyanotrimethylenecyclopropane (CN_6_–CP) exhibits the largest EA of 5.87 eV (estimated from cyclic voltammetry) which enables efficient doping of conjugated polymers with large IP.^[^ ^12^, ^13^, ^14^ ^]^ Although this mainstream design strategy has succeeded in developing extraordinarily strong dopants, we face inevitable three major issues for further development of such dopants which are i) the limitation in molecular design, ii) synthetic inaccessibility, and iii) chemical instability. The limitation in molecular design comes from the fact that dense introduction of the electron‐withdrawing group is far more effective to increase the EA of the dopant than extending the conjugation which leads to the ultimately simplified and nonextendable structure of CN_6_–CP. As the EA of dopant increases through design, it becomes increasingly difficult to obtain the neutral state through chemical synthesis because these compounds are energetically more stable at their charged state. Indeed, it takes a very strong oxidant such as thallic trifluoroacetate or nitrosonium salt to neutralize the anionic state of CN_6_–CP and requires proper synthetic skills and equipment to handle with.^[^ ^13^, ^14^ ^]^ Apart from the design and synthetic limitations, independent of its EA, the densely introduced electron‐withdrawing groups would undoubtfully raise the reactivity of the neutral dopants. For example, tetracyanoethylene (TCNE) possesses relatively small EA (4.95 eV)^[^ ^15^ ^]^ yet is known to be quite reactive toward nucleophilic attack because of its localized and highly positive charge (**Figure** **1**a).^[^ ^16^ ^]^ Such kind of nucleophilic attack can be suppressed by extending the *π*‐conjugation as is evidenced by the lower reactivity of TCNQ compared to TCNE despite having similar EA (4.91 eV).^[^ ^15^, ^17^ ^]^ Nonetheless, once the reactivity is enhanced, they can no longer be guaranteed to dope the target compound without any side reaction. Also, the increase in EA leads to the chemical instability of the dopant to certain atmospheres where the doping ability would be significantly reduced. Particularly, strong dopants with EA over 5.3 eV becomes overly sensitive to moisture where oxidation of H_2_O occurs.^[^ ^18^, ^19^ ^]^ In fact, some of the strong dopants are reported to deteriorate rapidly when exposed to atmospheric conditions and need to be stored under a dry and inert atmosphere once they have been synthesized.^[^ ^10^, ^14^ ^]^ From a practical viewpoint, these concerns lead to poor reproducibility and discourage the use of such compounds in industry.

From these circumstances, 2,3,5,6‐tetrafluoro‐7,7,8,8‐tetracyanoquinodimethane (F_4_–TCNQ) is the most widely adopted molecule among the large variety of molecular dopants that have been investigated.^[^ ^20^, ^21^ ^]^ As well as its commercial availability, F_4_–TCNQ is known as a chemically stable compound that is less vulnerable to nucleophilic attack because of its extended *π*‐conjugation and engages solely in electron transfer with its fairly large EA (5.28 eV)^[^ ^15^ ^]^ yet this driving force is still smaller than the redox potential of H_2_O which brings about good air stability. However, consequently, the doping level that could be achieved with F_4_–TCNQ was always limited by the energy offset between the IP of the host semiconducting polymer and the EA of the dopant under the conventional doping paradigm.^[^ ^22^ ^]^ To overcome this, we have demonstrated a new doping technique of semiconducting polymers so‐called anion exchange doping where the redox potential limitation of F_4_–TCNQ could be overcome in the presence of an external counter anion.^[^ ^23^ ^]^ The anion exchange of F_4_–TCNQ radical anion to external anion produces a gain in Gibbs free energy and facilitates the doping reaction beyond the limit of the energy offset between the IP of the host semiconducting polymer and EA of F_4_–TCNQ. As a result, a phenomenal carrier density of one hole per monomer unit with significantly improved crystallinity was achieved when using poly(2,5‐bis(3‐tetradecylthiophen‐2‐yl)thieno[3,2‐*b*]thiophene) (PBTTT–C14) as the host semiconducting polymer. The discovery of such unique doping phenomena paved the way for a paradigm shift in the design concept of an efficient organic dopant described as a binary system consisting of an initial doping component and counterion for neutralizing the doped polymer.

By feeding back the notion of the anion exchange doping system, we recently reported a new molecular dopant (tris(4‐bromophenyl)aminium bis(trifluoromethylsulfonyl)imide; TBPA–TFSI) where the triarylaminium radical cation (TBPA^•+^) serves as the initial doping component and TFSI anion (TFSI^−^) to compensate the charges of the doped semiconductor (Figure 1b).^[^ ^24^ ^]^ As represented by TBPA–SbCl_6_ (i.e., tris(4‐bromophenyl)aminium hexachloroantimonate), TBPA^•+^ is known as a persistent radical cation and widely adopted as a strong and clean oxidant.^[^ ^25^, ^26^, ^27^ ^]^ In addition, TFSI^−^ was chosen as the counter anion because the physicochemical properties, such as binding preference and hydrophobicity of TFSI^−^ significantly contribute to the stabilization of the conducting polymer system.^[^ ^23^ ^]^ Doping PBTTT–C14 with TBPA–TFSI resulted in the same or slightly higher doping level to anion exchange doping with unprecedented crystal structure where dissipation of the specific higher‐order diffraction could be observed because of the periodic formation of supramolecular cocrystals. As a result of such high carrier density with high crystallinity, electrical conductivity surpassed the value achieved with anion exchange doping which assessed TBPA–TFSI as a powerful dopant. Although TBPA–TFSI was stable in its solution state, we discovered that a substantial amount of TBPA–TFSI converted into dicationic salt of tetraaryl benzidine (TAB) (*N* ^4^,*N* ^4^,*N* ^4'^,*N* ^4'^‐tetrakis(4‐bromophenyl)‐[1,1'‐biphenyl]‐4,4'‐diammonium di{bis(trifluoromethylsulfonyl)imide}; TAB–2TFSI) through significantly fast disproportionation upon storage in its solid state (Figure 1b). There have been several reports on the disproportionation of TBPA^•+^ in literature and it has been implied that the rate depends on the energetic stability of TAB salts.^[^ ^25^, ^28^ ^]^ Because the spontaneous dimerization of TBPA–TFSI occurs rapidly, this prompted us to seek the use of the product as a stable dopant. Here, we demonstrate that the naturally‐derived TAB–2TFSI exhibits remarkable atmospheric stability and can be synthesized in gram scale without the use of special technique or equipment yet possessing the same level of redox potential as TBPA–TFSI. Doping PBTTT–C14 with TAB–2TFSI resulted in high doping level together with excellent crystallinity leading to a conductivity of 656 S cm^−1^. Through these findings, we aim to highlight salts composed of dicationic species as a strong and atmospherically stable p‐dopant that paves the way for its use not only for doping but also for various oxidation reactions with high reproducibility.

## 2 Result and Discussions

### 2.1 Disproportionation of TBPA–TFSI

**Figure 2:**
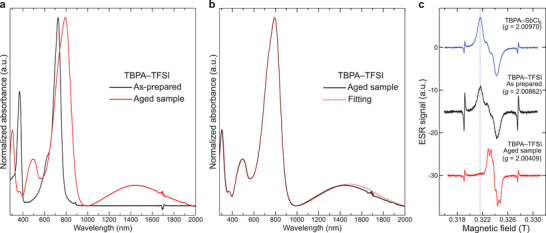
a) UV–vis–NIR absorption spectra of as‐prepared and aged TBPA–TFSI samples in CH_2_Cl_2_. b) Absorption spectra of aged TBPA–TFSI and reproduced spectra using authentic TBPA, TBPA–TFSI, TAB–TFSI, and TAB–2TFSI in CH_2_Cl_2_. c) ESR spectra of TBPA–SbCl_6_ as prepared and aged TBPA–FSI recorded at −73 °C in CH_2_Cl_2_. The antiphase signal observed in all samples corresponds to the Mn^2+^/MgO marker. Dash line indicates the residual TBPA^•+^ in the aged sample.

Although TBPA–SbCl_6_ is known as a stable *π*‐radical compound owing to the high donor ability of nitrogen atom,^[^ ^25^, ^26^, ^29^ ^]^ the radical cation decomposes to its neutral state over time in contact with atmospheric moisture. Besides, this compound is also infamous for disproportioning to its dimeric species which complicates spectroscopic measurements.^[^ ^28^, ^30^, ^31^ ^]^ However, such bleaching of radical cation and disproportionation takes place over several weeks to months when stored in its solid state under atmospheric conditions. Therefore, we were initially under the impression that TBPA–TFSI would show comparable or greater stability since the only chemical difference against TBPA–SbCl_6_ would be the counter anion and it is reported that larger counter anions that hinder the approach of radical cation species show little sign of disproportionation.^[^ ^25^, ^28^ ^]^ Contrary to our expectation, after a week of storage in its solid state under atmospheric condition (in a light‐shielding vial), acrid fuming was observed when the sample was taken out from the stored vial indicating a high decomposition rate of TBPA–TFSI (named aged sample hereafter). In stark contrast to the initial ultraviolet–visible–near‐infrared (UV–vis–NIR) absorption feature showing a sharp peak around 360 and 725 nm, the aged sample showed four rather broad absorption peaks at around 300, 490, 790, and 1445 nm (**Figure** **2**a). Aside from the absorption peak corresponding to the neutral TBPA at 300 nm, the newly observed peaks completely matched with the progressive absorption observed in an aged TBPA−SbCl_6_ reported by Rathore and others.^[^ ^30^ ^]^ The peaks at 490 and 1445 nm correspond to the radical cation absorption of TAB (TAB^•+^), while the absorption peak at 790 nm corresponds to the dication state of TAB (TAB^2+^). The existence of each species in the aged sample was also confirmed by the absorption spectrum of an authentic sample prepared separately (see Figure S2, Supporting Information). Using these data with the reported molar extinction coefficient of each species, the absorption spectrum of the aged sample including the slight shoulder peak at around 760 nm could be well reproduced (Figure 2b).^[^ ^30^, ^32^ ^]^ Surprisingly, the amount of remaining TBPA^•+^ was estimated to be only 9% in the aged sample and most of the TBPA–TFSI had been consumed to generate TBPA, TAB^•+^, and TAB^2+^ (33%, 25%, and 33%, respectively). Such a high conversion rate together with the correspondence between the actual and reproduced absorption spectrum of the aged sample indicates that the preconceived decomposition of TBPA–TFSI was a rapid and smooth disproportionation that predominantly produces TAB–TFSI and TAB–2TFSI. It should be noted that the disproportionation of TBPA–TFSI cannot be suppressed by the surrounding environment as the same behavior was observed for the sample stored in the N_2_ filled glovebox. Needless to say, the previously reported experimental data describing the doping ability of TBPA–TFSI was obtained from a freshly prepared compound in its solution state where the formation and effect of TAB species are negligible.^[^ ^24^ ^]^

The disproportionation of TBPA–TFSI was also confirmed by electron spin resonance (ESR) measurement (Figure 2c). The solution ESR signal of the fresh sample showed a nearly identical pattern to TBPA–SbCl_6_ (*g* = 2.00 970) with a *g* value of 2.00 862 because both compounds share the same TBPA^•+^. On the other hand, the aged sample showed a narrow ESR signal with a smaller *g* value of 2.00 409. The small peak in the baseline of the aged sample matches well with the peak top position of TBPA–SbCl_6_ and TBPA–TFSI indicating the presence of residual TBPA^•+^ in the aged sample which is consistent with the absorption spectrum. The narrow ESR spectrum and smaller *g* value of the aged sample indicate that the observed spin is more delocalized and being less affected by the heavy bromine atom which both strongly indicate the formation of TAB structure. Note that the ESR signal observed in the aged sample corresponds to TAB^•+^ (in TAB–TFSI) as the spinless TAB^2+^ (in TAB–2TFSI) remains silent in this measurement. Another interesting aspect of this disproportionation is its concentration‐dependent rate. While the diluted solution of TBPA–TFSI with a concentration in the order of 10^−5^ m showed identical absorption spectra upon storage up to 1 week, progressive absorption of the dimerized species was observed for a more concentrated solution (order of 10^≈2^ m) and this absorption peaks intensified after solidifying this solution (see Figure S3, Supporting Information). Such a fast‐rate disproportionation behavior upon solidification was also observed in solid‐state ESR measurement. The solid sample obtained by solidifying the as‐prepared TBPA–TFSI solution used for the solution‐state ESR measurement showed a *g* value of 2.0042 which was different from its solution state but nearly identical to the aged sample (*g* = 2.0040 in solid state).

Although we do not have concrete evidence, the disproportionation of TBPA–TFSI is most likely to happen similar to TBPA^•+^ derivatives through three steps which are 1) localization of the radical at the bromo‐substituted carbon of TBPA unit, 2) *σ*‐dimerization, and 3) elimination of molecular bromine.^[^ ^28^ ^]^ The acrid fuming is expected to be hydrobromic acid generated when the eliminated bromine contacts the atmospheric moisture. We emphasize that while the initial absorption spectrum of TBPA–SbCl_6_ is predominantly retained after a year of storage in its solid state under ambient atmosphere, TBPA–TFSI had almost lost its absorption feature in a week. This striking difference demonstrates that the disproportionation of TBPA^•+^ strongly depends on the counter anion that defines its aggregation structure. Because of its rapid disproportionation behavior, several attempts to obtain the single crystal of TBPA–TFSI yielded the single crystal of TAB–2TFSI which impeded the further investigation of the origin of such disproportionation.

### 2.2 Synthesis of TAB–2TFSI

**Figure 3:**
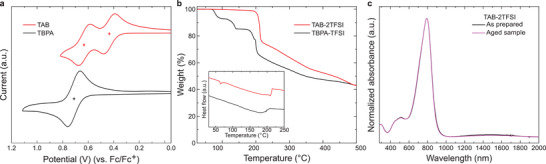
a) Cyclic voltammograms of TAB and TBPA in CH_2_Cl_2_ at a concentration of 1 × 10^−3^ m. Crosses indicate the half‐wave oxidation potentials of each compound. b) TG curves of TAB–2TFSI and TBPA–TFSI. DTA curves of both compounds are shown in the inset. c) UV–vis–NIR spectra in CH_2_Cl_2_ of as prepared TAB–2TFSI and the same sample stored in a transparent vial under atmospheric condition for one year.

As described in several literature, the stability of TBPA^•+^ depends on the repression of the formation of TAB^•+^ and TAB^2+^ via debromination.^[^ ^28^, ^30^, ^31^ ^]^ In other words, the disproportionation of TBPA^•+^ accelerates if the stability of the final product is high. The fact that TBPA–TFSI converts to TAB species at an exceptionally fast rate among the variety of TBPA^•+^ salts reported so far strongly encouraged us to investigate the use of these compounds as highly stable dopants. To estimate the doping ability of the two compounds, we synthesized the neutral TAB (see the Supporting Information) and conducted cyclic voltammetry (CV) measurement in comparison with neutral TBPA. As shown in **Figure** **3**a, the driving force for p‐type doping (formal potential) of the TAB^2+^ was comparable to TBPA^•+^ as can be seen from the 2nd half‐wave oxidation potential of TAB in the cyclic voltammogram matching with the 1st half‐wave oxidation potential of TBPA, with values of 0.63 and 0.70 V against ferrocene, respectively. Assuming the ferrocene/ferrocenium redox potential as −5.1 eV against vacuum level,^[^ ^33^ ^]^ these values correspond to maximum doping ability up to −5.80, and −5.73 eV for TBPA^•+^ and TAB^2+^, respectively. On the other hand, the formal potential of TAB^•+^ was quite small compared to the above two species (1st half‐wave oxidation potential of TAB; 0.43 V against ferrocene). Nonetheless, the doping ability of TAB^•+^ can reach −5.53 eV, which is greater than F_4_–TCNQ.^[^ ^15^ ^]^ Because the main research focus is on the development of strong and stable dopants, the material of interest will mainly be TAB–2TFSI hereafter.

The synthesis of TAB–2TFSI was carried out following the same procedure as TBPA–TFSI (Scheme S1, Supporting Information).^[^ ^34^ ^]^ TAB was smoothly oxidized to TAB^2+^ and converted to TAB–2TFSI in the presence of AgTFSI in high yield at a gram scale (see the Supporting Information for detailed procedure). One noticeable difference between TBPA–TFSI and TAB–2TFSI during the synthetic process is their sensitivity to atmospheric moisture. Since the purity and yield of TBPA^•+^ salts are known to be substantially moisture dependent, the workup and purification processes for TBPA–TFSI are required to be done rapidly and (or) under an inert atmosphere. On the other hand, TAB–2TFSI could be isolated in high yield through open‐air filtration and precipitation which is advantageous for mass production.

### 2.3 Stability of TAB–2TFSI

The stability of chemical compounds can be assessed from variable aspects. For molecular dopants, the value can be found from the combination of thermal and atmospheric stability because handling strong dopants in their solid state regardless of the surrounding atmosphere would expand the versatility of their use with high reproducibility. To date, there have been very few reports on the thermal stability of the dopant itself. This is because strong dopants are generally unstable under ambient conditions which discourage the investigations on their thermal properties. Fortunately, TAB–2TFSI was stable enough to be handled during the thermal analysis. The thermal stability of TAB–2TFSI was demonstrated by thermal gravimetric (TG) measurement and differential thermal analysis (DTA). Compared to freshly prepared TBPA–TFSI that shows decomposition below 100 °C (5% weight loss temperature: *T*_95_ = 84 °C), TAB–2TFSI exhibited significantly higher *T*_95_ of 206 °C, with an endothermic transition around 65 °C which was confirmed as a different crystal phase transition as no melting behavior was observed during the melting point measurement (Figure 3b). The air stability of TAB–2TFSI was evaluated by comparing the absorption spectrum of the as‐prepared sample and aged sample which was stored in a vial under ambient atmosphere for one year. As anticipated, no trace of decomposition was observed after the compound was exposed to atmospheric conditions for a prolonged period and demonstrated the excellent stability of TAB–2TFSI (Figure 3c). Aside from disproportionation, neutralization of the radical cation generally occurs in the TBPA^•+^ system when in contact with water which is why technical workup and purification are required. In this context, because of the excellent air stability of TAB–2TFSI, the contact with atmospheric moisture does not neutralize this dopant, which allows scalable open‐air synthetic procedures as described above. Although some dopants have been reported to exhibit high thermal stability,^[^ ^10^, ^14^ ^]^ to the best of our knowledge, this is the first report of a strong p‐dopant with high thermal stability as well as excellent atmospheric stability.

**Figure 4:**
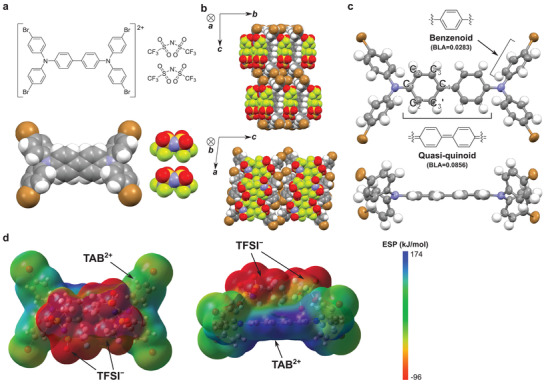
a) Chemical structure of TAB–2TFSI and corresponding space‐filling model extracted from crystal structure data. b) Crystal structure of TAB–2TFSI in the space‐filling model and c) top and side view of the conformation of TAB unit within the crystal structure in the ellipsoid model with probability level at 50% excepting H atoms. The bond‐length alternation (BLA) is defined as the difference between the average distance of C_1_–C_2_, C_1_–C_2_’, C_3_–C_4_, C_3_’–C_4_, and the average distance of C_2_–C_3_, C_2_’–C_3_’. d) Top and side view of ESP map of TAB–2TFSI calculated from the crystal structure at the UM062X/6‐31++G (d,p) level of theory.

Although the stability of TAB–2TFSI is unimpeachable, this is certainly inconsistent with the redox reaction against H_2_O. Because TAB–2TFSI was estimated to exhibit a larger redox potential than it is required to oxidize H_2_O, it is hard to imagine this compound to be indefinitely stable under exposure to atmospheric conditions. One might think that the actual redox potential of TAB^2+^ is quite small compared to the estimated value derived from the CV data of the neutral TAB described in Section 2.2 (Figure 3a). However, the CV measurement of TAB–2TFSI confirms that the redox potential of TAB^2+^ is consistent with the estimated value as shown in Figure S4 (Supporting Information). To further gain insight into the origin of such high atmospheric stability, a single‐crystal analysis of TAB–2TFSI was performed. TAB^2+^ and TFSI^−^ were aggregated in an alternate‐stack form in which the two TFSI^−^s were positioned on top of the central biphenyl unit of TAB (**Figure** **4**a,b). The bond‐length alternation (BLA) values of the central biphenyl unit and the flanked bromo‐substituted benzene rings were calculated to be 0.0856 and 0.0283, respectively. These values can be translated to the quasiquinoid structure of the biphenyl unit and benzenoid structure of the flanked benzene rings and this strongly suggests that TAB–2TFSI features a dicationic nature.^[^ ^35^, ^36^ ^]^ The quasiquinoidal form of the biphenyl unit is also indicated by the planar structure shown in Figure 4c. Unlike TBPA–TFSI, the disproportionation can be completely restrained in TAB–2TFSI because the closed‐shell cation does not allow the *δ*‐dimerization which largely differentiates the two charged compounds. From the data shown above, the positive charges of TAB are most likely delocalized over the central biphenyl unit and are surrounded by TFSI^−^. From the ESP map calculated from the crystal structure, indeed, the highly positive charge is confined within the biphenyl unit and is completely shielded by the negative charge of TFSI^−^ (Figure 4d). We attribute this aggregation structure to the high atmospheric stability of TAB–2TFSI because the hydrophobic TFSI^−^ serves as an encapsulator of the positive charges that are responsible for the redox reaction. Although the above discussion on the relationship between the aggregation structure and atmospheric stability of TAB–2TFSI is based on the single crystal analysis, we strongly believe that the same principle can be applied to the actual synthesized product as the powder X‐ray diffraction (PXRD) data show good agreement with the simulated PXRD patterns from the single crystal analysis data (see Figure S5, Supporting Information). Accordingly, this strongly implies the advantage of the charged molecule over the neutral molecule as a strong and atmospherically stable dopant. As the charged molecule consists of two ionic species, the strength and stability can be separately tuned which significantly extends the versatility of its chemical design. For instance, for p‐dopants, the redox potential of the cationic species can be increased by introducing an electron‐withdrawing group while the charge delocalization can be confined within a certain unit. Therefore, the charge can be stabilized by extending the conjugation length or introducing bulky substituents to this unit. In concert with this, the charged state of cationic species can be stabilized by the counter anion as is the case with TAB–2TFSI. This stabilization not only originates from the hydrophobic nature of the counter anion but also from the aggregation structure that leaves no cavity for water or oxygen molecules to approach.^[^ ^37^ ^]^ Also, the hard and soft acids and bases (HSAB) theory can potentially be applied to accelerate the stability of dopants.^[^ ^38^ ^]^ Because aqueous media in which hard ion is involved plays a critical role in the deterioration of strong p‐dopant upon exposure to atmospheric conditions, increasing the softness of the dopant's ion pair will energetically disfavor the deteriorating reaction. In our previous report on the anion exchange doping, indeed, the physicochemical property of the counter anion dramatically affected the thermal durability of the doped polymer system which strongly supports this hypothesis.^[^ ^23^ ^]^

### 2.4 Evaluation of TAB–2TFSI as a Strong Dopant

**Figure 5:**
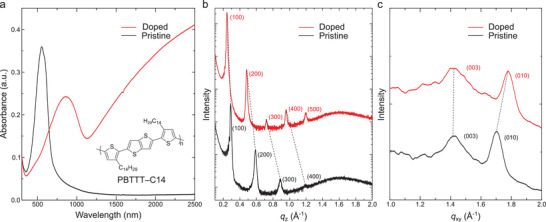
a) UV–vis–NIR absorption spectra of pristine and doped PBTTT–C14 thin film. Inset shows the chemical structure of PBTTT–C14. b) Out‐of‐plane and c) in‐plane XRD diffraction data of pristine and doped PBTTT–C14 thin film.

The p‐type doping ability of TAB–2TFSI was tested using PBTTT–C14 as the host semiconducting polymer (methods are described in the Supporting Information). When PBTTT–C14 film was immersed in a 1.5 × 10^−3^ m acetonitrile solution of TAB–2TFSI, a drastic color change was observed. The UV–vis–NIR absorption spectrum of the doped film showed total bleaching of the absorption band corresponding to the neutral polymer around 500 nm. The additional peak around 900 nm and a broad absorption band extending over the entire NIR region correspond to polaron and delocalized free carrier, respectively (**Figure** **5**a). The fact that the absorption spectrum of the TAB–2TFSI doped PBTTT–C14 was identical to our previous reports strongly suggests that very high carrier density (close to one hole per monomer unit) was achieved.^[^ ^23^, ^24^ ^]^ The impact of the doping process on the microstructure ordering of PBTTT–C14 was also confirmed by X‐ray diffraction (XRD) measurement (Figure 5b,c). A noticeable expansion of lamella stacking and shrinking of *π*–stacking can be seen from the shift of out‐of‐plane (*h*00) and in‐plane (010) diffraction peaks, respectively. The expansion of lamella stacking distance originates from the storage of TFSI^−^ within the lamella structure and the shrinking of *π*–stacking is probably due to the improved planarity of the backbone^[^ ^23^ ^]^ or the coulombic attraction between TFSI^−^ and positively charged polymer chain.^[^ ^39^ ^]^ The degree of crystallinity was also prominently enhanced after doping as can be seen from the decrease of the full width at half maximum (FWHM) of (100) diffraction peak and the appearance of higher‐order lamella diffraction up to (500) diffraction. Also, the progression of the FWHM of each (*h*00) diffraction (*δ*FWHM) was greatly decreased indicating the long‐range order with suppressed cumulative disorder throughout the doped film (see Figure S6, Supporting Information).^[^ ^34^, ^40^ ^]^ Furthermore, a significant decrease in the intensity of (300) diffraction peak was observed in the doped film which originates from the formation of cocrystals between doped polymers and counter anions. This unique XRD profile suggests that the weighted‐center position and number of TFSI^−^ is precisely confined against the repeating unit of PBTTT–C14,^[^ ^24^ ^]^ which again supports the high doping level estimation from the absorption data. From the 2‐terminal conductivity measurement, the doped film exhibited a high electrical conductivity of 656 S cm^−1^ which is consistent with the result obtained by using TBPA–TFSI. To further precisely confirm the consistency between the TAB–2TFSI doped PBTTT–C14 and our previous reports in terms of carrier mobility and density, Hall effect measurement was conducted. As shown in Figure S8 (Supporting Information), a clear Hall voltage response against the applied magnetic field was observed which implies the coherent carrier transport in the doped PBTTT–C14 film. The Hall carrier density and Hall mobility derived from the standard expression of the Hall effect were ≈1.9 × 10^21^ cm^−3^ and 1.6 cm^2^ V^−1^ s^−1^, respectively. The calculated carrier density corresponds to one hole per monomer unit or slightly higher which agrees well with our previously reported values using anion exchange doping or TBPA–TFSI.^[^ ^23^, ^24^ ^]^ All the above‐mentioned striking impact of p‐type doping using TAB–2TFSI on the physical, structural, and electronic properties of PBTTT–C14 thin film were in excellent agreement with our previous report using anion exchange doping method or TBPA–TFSI, and demonstrates efficient and strong doping capability of TAB–2TFSI.

With the excellent stability of TAB–2TFSI, we envisage the use of a highly concentrated dopant solution for the doping process that is suitable for screening experiments. To demonstrate this, we performed a spin‐cast doping procedure where a highly concentrated TAB–2TFSI solution (100 × 10^−3^ m) was dropped on top of a spinning PBTTT–C14 thin film. Owing to the high concentration of the dopant solution and the large energy offset between the IP of PBTTT–C14 (4.80 eV) and the redox potential of TAB–2TFSI (5.73 eV), a similar doping level was achieved within an extremely short time scale (few seconds) evidenced by the absorption spectra and XRD data (Figure S9, Supporting Information). As a result, spin‐cast doped PBTTT–C14 exhibited an electrical conductivity of 630 S cm^−1^ which is perfectly consistent with the result from the conventional doping procedure (656 S cm^−1^). Such doping procedure can only be done using highly stable dopants and would potentially accelerate the screening experiment for developing high‐performance conducting polymers.

### 2.5 Doping Mechanism of TAB–2TFSI

**Figure 6:**
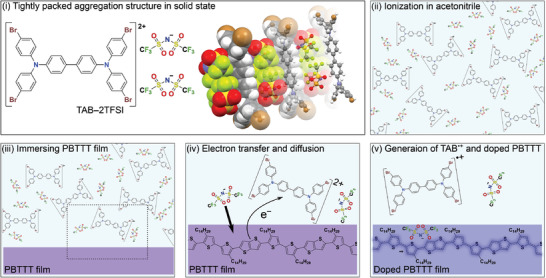
Proposed doping mechanism of TAB–2TFSI. The doping process described in (iv) and (v) is the magnified images of the dotted square region in (iii). The blurred part of PBTTT–C14 in (v) depicts the delocalized hole along the backbone.

Once again, from the conventional understanding of strong dopants, the stability and p‐doping ability of TAB–2TFSI are contradictory. Here, based on the presented data, we attempt to draw a proposed doping mechanism of TAB–2TFSI (**Figure** **6**). Upon dissolving TAB–2TFSI in acetonitrile, the tightly packed aggregation structure i) can be dissociated to rather naked TAB^2+^ and TFSI^−^ ii). When PBTTT–C14 thin film is immersed in this solution, TAB^2+^ that is responsible for doping can easily approach the surface of PBTTT–C14 iii). Because of the large energy offset between the IP of PBTTT–C14 and redox potential of TAB^2+^ (> 0.9 eV), immediate electron transfer from PBTTT–C14 to TAB^2+^ occurs producing TAB^•+^ and hole (iV and V). Both positively charged species are counterbalanced with TFSI^−^, which means that one of the TFSI^−^ remains in the solution while the other TFIS^−^ diffuses into the PBTTT–C14 film. Because the redox potential of TAB^2+^ is fairly larger than that of TAB^•+^ (the difference is 0.2 eV) and the doping experiment is carried out using an excess amount of TAB–2TFSI, TAB^2+^ is preferentially consumed in the doping process and the produced TAB^•+^ remains in the solution. In addition, because the diffusion of TFSI^−^ only takes place to compensate the hole in doped PBTTT–C14, the TAB unit does not diffuse in the doped film regardless of its charged states. To verify this mechanism, the components in the dopant solution and PBTTT–C14 thin film before and after doping were evaluated by absorption spectrum and X‐ray photoelectron spectroscopy (XPS), respectively. As shown in Figure S10a (Supporting Information), additional absorption bands around 480 and 1330 nm that are characteristic of TAB^•+^ were observed in the dopant solution after doping PBTTT–C14. From this data, it is undoubtful that the amount of TAB^•+^ is increasing in the dopant solution after the doping process. Meanwhile, as for the PBTTT–C14 thin film, a clear change in the atomic composition was observed before and after doping in the elemental analysis result from XPS measurement (Figure S10b, Supporting Information). While the undoped film only showed carbon and sulfur atom signals, additional signals corresponding to fluorine, oxygen, and nitrogen atoms were observed in the doped film which were identically observed TAB–2TFSI. On the other, no trace of peaks corresponding to bromine atom in TAB–2TFSI were observed in the doped film which indicates the absence of any TAB unit in the doped film. Both results were in perfect agreement with our hypothesis and strongly support our proposed doping mechanism of TAB–2TFSI.

## 3 Conclusion

In summary, we have coincidently discovered that the previously reported strong dopant TBPA–TFSI spontaneously converts to TAB–TFSI and –2TFSI through rapid debromodimerization in the solid state. Contrarily, this provided a strong message that the major products would be extremely stable as no other TBPA^•+^ salts disproportionate at this rate. By evaluating the doping ability of TAB species, it was found that the TAB–2TFSI dopant can be as strong as TBPA–TFSI. The two‐step synthesis of TAB–2TFSI was performed in high yield using standard precipitation and filtration technique which is advantageous for mass production. The synthesized TAB–2TFSI exhibited good thermal stability and excellent stability that showed no trace of deterioration upon storage under atmospheric conditions for more than 1 year. Single crystal analysis revealed that TAB–2TFSI represented a dicationic form in which the charges responsible for the strong redox reaction are delocalized among the central biphenyl unit and is tightly surrounded by counter TFSI^−^. The structural information of TAB–2TFSI implies that both delocalization of cationic charges and packing motif of hydrophobic TFSI^−^ contribute to its excellent atmospheric stability. Despite such high stability, a very high doping level when doping PBTTT–C14 was confirmed by absorption, XRD, conductivity, and Hall effect measurements. Moreover, the high stability of TAB–2TFSI enabled the use of a highly concentrated dopant solution which allowed spin‐cast doping process with identical results to conventional doping procedure. The data provided here highlights the charged molecule as a new class of strong dopants that is synthetically accessible, versatile in chemical design, exceptionally stable, and possesses high doping capability that guarantees high reproducibility.

## 4 Experimental Section

### Synthesis of *N* ^4^,*N* ^4^,*N* ^4'^,*N* ^4'^–Tetrakis(4–bromophenyl) –[1,1'–biphenyl]–4,4'–diammonium di{bis(trifluoromethylsulfonyl)imide} (TAB‐2TFSI)

A suspension containing TAB (804 mg, 1.00 mmol) and AgTFSI (1.16 g, 3.00 mmol) in ether (80 mL) was cooled to 0 °C, and to this was added dropwise a solution of iodine (761 mg, 3.00 mmol) in ether (20 mL) over 10 min. The suspension immediately changed its color from white to orange and to dark green as the addition of iodine proceeded. After the addition was completed, the mixture was stirred for an additional 30 min and the brown precipitate which is the mixture of AgI and the target compound was collected by suction filtration. The solid was dispersed in 300 mL of CH_2_Cl_2_ and the mixture was passed through Celite to remove AgI. Ether (300 mL) was added to the filtrate and the precipitate was collected by suction filtration to obtain the title compound 1.18 g in 87% yield as a wine‐red microcrystal. No signal could be observed in ^1^H‐ and ^13^C‐NMR due to the trace amount of radical species that was generated by the residual water in the deuterated solvent. ^19^F‐NMR (376 MHz, CD_2_Cl_2,_ *δ*): −78.0 (s, 12F, —CF_3_ of TFSI^−^). Anal. calcd. For C_40_H_24_Br_4_F_12_N_4_O_8_S_4_: C 35.21, H 1.77, N 4.11. Found: C 35.01, H 1.93, N 4.12. Block‐shaped wine‐red single crystals were grown from the vapor‐diffusion method using CH_2_Cl_2_ and ether under ambient atmosphere. [CCDC 2074406 contains the supplementary crystallographic data for this paper. These data can be obtained free of charge from The Cambridge Crystallographic Data Centre via www.ccdc.cam.ac.uk/data_request/cif.]

### Evaluation of Electrical Conductivity of Doped PBTTT–C14

The conductivity of doped PBTTT–C14 was evaluated from 2 terminal device with precisely patterned channel length and width. The sheet conductance was determined by taking the slope of the linear fitted current–voltage curve measured using KEITHLEY 2400 source meter and the conductivity was obtained by dividing the sheet conductance by the average film thickness. Detail device fabrication procedures are described in the Supporting Information.

## Conflict of Interest

The authors declare no conflict of interest.

## Supporting information

Supporting Information

## Data Availability

Research data are not shared.

## References

[1] T. Ito , H. Shirakawa , S. Ikeda , J. Polym. Sci., Polym. Chem. Ed. 1974, 12, 11.

[2] H. Shirakawa , E. J. Louis , A. G. MacDiarmid , C. K. Chiang , A. J. Heeger , J. Chem. Soc. Chem. Commun. 1977, 578. 10.1039/c39770000578.

[3] C. K. Chiang , C. R. Fincher , Y. W. Park , A. J. Heeger , H. Shirakawa , E. J. Louis , S. C. Gau , A. G. MacDiarmid , Phys. Rev. Lett. 1977, 39, 1098.

[4] I. E. Jacobs , A. J. Moulé , Adv. Mater. 2017, 29, 1703063. 10.1002/adma.201703063 28921668

[5] X. Guo , A. Facchetti , Nat. Mater. 2020, 19, 922. 32820293 10.1038/s41563-020-0778-5

[6] J. G. Ibanez , M. E. Rincón , S. Gutierrez‐Granados , M. Chahma , O. A. Jaramillo‐Quintero , B. A. Frontana‐Uribe , Chem. Rev. 2018, 118, 4731. 29630346 10.1021/acs.chemrev.7b00482

[7] T. Nezakati , A. Seifalian , A. Tan , A. M. Seifalian , Chem. Rev. 2018, 118, 6766. 29969244 10.1021/acs.chemrev.6b00275

[8] I. Salzmann , G. Heimel , M. Oehzelt , S. Winkler , N. Koch , Acc. Chem. Res. 2016, 49, 370. 26854611 10.1021/acs.accounts.5b00438

[9] H. Méndez , G. Heimel , A. Opitz , K. Sauer , P. Barkowski , M. Oehzelt , J. Soeda , T. Okamoto , J. Takeya , J.‐B. Arlin , J.‐Y. Balandier , Y. Geerts , N. Koch , I. Salzmann , Angew. Chem., Int. Ed. 2013, 52, 7751. 10.1002/anie.201302396 23784880

[10] Z. Q. Gao , B. X. Mi , G. Z. Xu , Y. Q. Wan , M. L. Gong , K. W. Cheah , C. H. Chen , Chem. Commun. 2008, 117. 10.1039/b713566a 18399419

[11] P. K. Koech , A. B. Padmaperuma , L. Wang , J. S. Swensen , E. Polikarpov , J. T. Darsell , J. E. Rainbolt , D. J. Gaspar , Chem. Mater. 2010, 22, 3926.

[12] T. Fukunaga , J. Am. Chem. Soc. 1976, 98, 610.

[13] T. Fukunaga , M. D. Gordon , P. J. Krusic , J. Am. Chem. Soc. 1976, 98, 611.

[14] Y. Karpov , T. Erdmann , I. Raguzin , M. Al‐Hussein , M. Binner , U. Lappan , M. Stamm , K. L. Gerasimov , T. Beryozkina , V. Bakulev , D. V. Anokhin , D. A. Ivanov , F. Günther , S. Gemming , G. Seifert , B. Voit , R. Di Pietro , A. Kiriy , Adv. Mater. 2016, 28, 6003. 27172371 10.1002/adma.201506295

[15] The EA values of the commercially available compounds were estimated from cyclic voltammetry using ferrocene as the reference. See the Supporting Information for more detail.

[16] D. N. Dhar , Chem. Rev. 1967, 67, 611.

[17] M. Kivala , C. Boudon , J.‐P. Gisselbrecht , B. Enko , P. Seiler , I. B. Müller , N. Langer , P. D. Jarowski , G. Gescheidt , F. Diederich , Chem. – Eur. J. 2009, 15, 4111. 19266523 10.1002/chem.200802563

[18] A. J. Bard , R. Parsons , J. Jordan , Standard Potentials in Aqueous Solution, Routledge, Boca Raton, FL 2017.

[19] C. G. Tang , M. C. Y. Ang , K.‐K. Choo , V. Keerthi , J.‐K. Tan , M. N. Syafiqah , T. Kugler , J. H. Burroughes , R.‐Q. Png , L.‐L. Chua , P. K. H. Ho , Nature 2016, 539, 536. 27882976 10.1038/nature20133

[20] W. Gao , A. Kahn , Appl. Phys. Lett. 2001, 79, 4040.

[21] E. F. Aziz , A. Vollmer , S. Eisebitt , W. Eberhardt , P. Pingel , D. Neher , N. Koch , Adv. Mater. 2007, 19, 3257.

[22] K. Kang , S. Watanabe , K. Broch , A. Sepe , A. Brown , I. Nasrallah , M. Nikolka , Z. Fei , M. Heeney , D. Matsumoto , K. Marumoto , H. Tanaka , S. Kuroda , H. Sirringhaus , Nat. Mater. 2016, 15, 896. 27159015 10.1038/nmat4634

[23] Y. Yamashita , J. Tsurumi , M. Ohno , R. Fujimoto , S. Kumagai , T. Kurosawa , T. Okamoto , J. Takeya , S. Watanabe , Nature 2019, 572, 634. 31462795 10.1038/s41586-019-1504-9

[24] Y. Yamashita , J. Tsurumi , T. Kurosawa , K. Ueji , S. TsunedaYukina , H. Kohno , S. Kempe , T. Kumagai , J. Okamoto , S. Takeya , Watanabe , Commun. Mater. 2021, 2, 45.

[25] N. G. Connelly , W. E. Geiger , Chem. Rev. 1996, 96, 877. 11848774 10.1021/cr940053x

[26] M. Quiroz‐Guzman , S. N. Brown , Acta Crystallogr., Sect. C: Cryst. Struct. Commun. 2010, 66, m171. 10.1107/S0108270110019748 20603548

[27] A. I. Hofmann , R. Kroon , S. Zokaei , E. Järsvall , C. Malacrida , S. Ludwigs , T. Biskup , C. Müller , Adv. Electron. Mater. 2020, 6, 2000249.

[28] L. Eberson , B. Larsson , C. Moberg , K. D. Krautwurst , P. Krogsgaard‐Larsen , R. Ryhage , R. Isaksson , Acta Chem. Scand. 1986, 40b, 210.

[29] T. Nishinaga , K. Komatsu , Org. Biomol. Chem. 2005, 3, 561. 15703786 10.1039/b418872a

[30] M. R. Talipov , M. M. Hossain , A. Boddeda , K. Thakur , R. Rathore , Org. Biomol. Chem. 2016, 14, 2961. 26878458 10.1039/c6ob00140h PMC5102333

[31] T. A. Schaub , T. Mekelburg , P. O. Dral , M. Miehlich , F. Hampel , K. Meyer , M. Kivala , Chem. – Eur. J. 2020, 26, 3264. 31970834 10.1002/chem.202000355 PMC7154785

[32] S. Amthor , B. Noller , C. Lambert , Chem. Phys. 2005, 316, 141.

[33] C. M. Cardona , W. Li , A. E. Kaifer , D. Stockdale , G. C. Bazan , Adv. Mater. 2011, 23, 2367. 21462372 10.1002/adma.201004554

[34] F. A. Bell , A. Ledwith , D. C. Sherrington , J. Chem. Soc. C 1969, 2719. 10.1039/j39690002719.

[35] L. K. Montgomery , J. C. Huffman , E. A. Jurczak , M. P. Grendze , J. Am. Chem. Soc. 1986, 108, 6004. 22175364 10.1021/ja00279a056

[36] G. Tan , X. Wang , Acc. Chem. Res. 2017, 50, 1997. 28731693 10.1021/acs.accounts.7b00229

[37] Y. Hua , Y. Liu , C.‐H. Chen , A. H. Flood , J. Am. Chem. Soc. 2013, 135, 14401. 24028552 10.1021/ja4074744

[38] R. G. Pearson , J. Chem. Educ. 1968, 45, 581.

[39] I. Garcia‐Yoldi , J. S. Miller , J. J. Novoa , J. Phys. Chem. A 2009, 113, 7124. 19489553 10.1021/jp901930s

[40] R. Noriega , J. Rivnay , K. Vandewal , F. P. V. Koch , N. Stingelin , P. Smith , M. F. Toney , A. Salleo , Nat. Mater. 2013, 12, 1038. 23913173 10.1038/nmat3722

