# Supplementary material for: Strong and Atmospherically Stable Dicationic Oxidative Dopant
Source: Adv Sci (Weinh). 2021 Oct 28;8(24):2101998. doi: 10.1002/advs.202101998 (PMC8693046; doi:10.1002/advs.202101998)
Supplement: Supplementary file 1 — Supporting Information [file ADVS-8-2101998-s001.pdf]

WILEY-VCH

© 2021 Wiley-VCH GmbH

ADVANCED SCIENCE
Open Access

Supporting Information

for *Adv. Sci.*, DOI: 10.1002/advs.202101998

Strong and Atmospherically Stable Dicationic Oxidative Dopant

Tadanori Kurosawa, Toshihiro Okamoto*, Yu Yamashita, Shohei Kumagai, Shun Watanabe, and Jun Takeya*

Supporting Information

Strong and Atmospherically Stable Dicationic Dopant

Tadanori Kurosawa, Toshihiro Okamoto*, Yu Yamashita, Shohei Kumagai, Shun Watanabe, and Jun Takeya*

Experimental Procedures

Materials.

All reagents were used as received unless otherwise noticed. Tris(4-bromophenyl)amine (TBPA), *N*,*N*,*N*′,*N*′-tetraphenyl benzidine, silver bis(trifluoromethanesulfonyl)imide (AgTFSI) were purchased from Tokyo Chemical Industry Co., Ltd. Iodine, *N,N*-dimethylformamide (DMF), diethyl ether (ether), dichloromethane (CH_2Cl_2) were purchased from KANTO chemical Co., Ltd. The solvents were further purified with a Glass Contour solvent purification system prior to use. *N*-bromosuccinimide (NBS) and anhydrous acetonitrile were purchased from FUJIFILM Wako Pure Chemical Corporation. NBS was recrystallized from water at a concentration of 0.1 wt% prior to use. PBTTT–C14 was synthesized according to the previous report.[S1] Number average molecular weight and dispersity evaluated by high-temperature size exclusion chromatography (*o*-dichlorobenzene, 140 °C) were 28.9 kDa and 1.5, respectively.

General for Synthesis and Characterization.

Nuclear Magnetic Resonance (NMR) spectra were recorded in a JEOL ECS 400 spectrometer. Chemical shifts are reported in parts per million (ppm, *δ* scale) from the residual proton in deuterated solvents (*δ* 7.26 ppm from deuterated chloroform; $CDCl_3$). For ^{19}F NMR, hexafluorobenzene was used as the standard reference (*δ* -164 ppm). ESR data were acquired using a JEOL-X310 spectrometer. Each sample was dissolved in anhydrous CH_2Cl_2 at a concentration below 1 mM and charged into a quartz ESR tube. For solid-state measurement, CH_2Cl_2 was gently removed under vacuum in the ESR tube for sample preparation. All measurements were performed at −73 °C. The magnetic field and ESR intensity were calibrated with a standard Mn^{2+}/MgO marker. Melting point measurement was performed using Mettler Toledo MP70 Melting Point System. Elemental

analysis was carried out on a J-Science Lab JM10 CHN analyzer. The absorption spectra were obtained from a JASCO V-670 UV-VIS-NIR Spectrophotometer using either a quartz cuvette or glass substrate. Thermal gravimetric (TG) measurement and differential thermal analysis (DTA) were conducted on Rigaku Thermo plus EVO2 TG-DTA 8121 with a heating rate of 5 °C/min under 100 ccm N_2 flow. Cyclic voltammetry (CV) was recorded using BAS ALS622D electrochemical analyzer. Cyclic voltammograms were recorded in 0.1 M $[NBu_4][PF_6]$ CH_2Cl_2 solution (for TBPA and TAB), 0.1 M $[NBu_4][PF_6]$ acetonitrile solution (for TCNE, TCNQ, F_4–TCNQ, and TAB–2TFSI) at a scan rate of 100 mV/s with glassy carbon working electrode, Pt wire counter electrode, and a silver wire in 0.01 M $[Ag][NO_3]$ / 0.1 M $[NBu_4][PF_6]$ benzonitrile solution as a reference electrode. Single-crystal X-ray diffraction (XRD) data were collected on a Rigaku R-AXIS RAPID II imaging plate diffractometer with Cu*K*α radiation (λ = 1.54187 Å). Thin-film XRD and was recorded on a Rigaku SmartLab with Cu*K*α radiation. PXRD measurement was performed using Rigaku XtaLAB Synergy with Cu*K*α radiation. A Gandolfi camera was used to minimize the preferred orientation of the bulk powder sample. Crystallographic data of TAB–2TFSI has been deposited in the Cambridge Crystallographic Data Centre (CCDC). The data can be obtained free of charge from the CCDC via www.ccdc.cam.ac.uk/data_request/cif. XPS was performed using KRATOS ULTRA 2 with monochromatic Al *K*α X-ray.

Scheme S1. Synthesis of TAB–2TFSI.

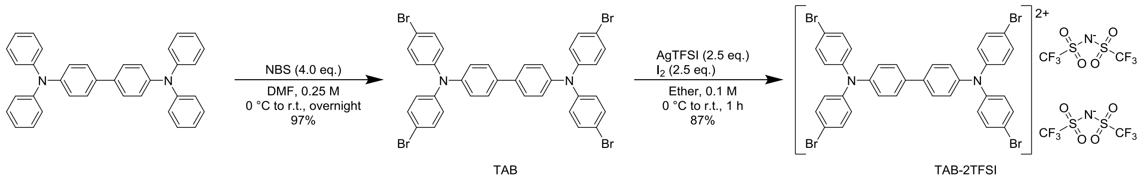

Synthesis of N^4,N^4,$N^{4'}$,$N^{4'}$–Tetrakis(4–bromophenyl)–[1,1'–biphenyl]–4,4'–diamine (TAB)

N-bromosuccinimide (7.12 g, 40.0 mmol) was added to a solution of *N*,*N*,*N'*,*N'*–tetraphenylbenzidine (4.89 g, 10.0 mmol) in DMF (70 mL), and the mixture was stirred overnight before being poured into MeOH. The precipitate was collected by suction filtration to give the title compound in 97% yield (7.82 g). No further purification was required as the crude compound showed an identical NMR spectrum to the reported data.[S2] ^{1}H-NMR (400 MHz, $CDCl_3$, δ) 7.45 (d, J = 8.2 Hz, 4H), 7.36 (d, J = 8.7 Hz, 8H), 7.10 (d, J = 8.7 Hz, 4H), 6.98 (d, J = 8.7 Hz, 8H).

Synthesis of N^4,N^4,$N^{4'}$,$N^{4'}$–Tetrakis(4–bromophenyl)–[1,1'–biphenyl]–4,4'–diammonium di{bis(trifluoromethylsulfonyl)imide} (TAB-2TFSI)

A suspension containing TAB (804 mg, 1.00 mmol) and AgTFSI (1.16 g, 3.00 mmol) in ether (80 mL) was cooled to 0 °C, and to this was added dropwise a solution of iodine (761 mg, 3.00 mmol) in ether (20 mL) over 10 min. The suspension immediately changed its color from white to orange and to dark

green as the addition of iodine proceeds. After the addition was completed, the mixture was stirred for an additional 30 min and the brown precipitate which is the mixture of AgI and the target compound was collected by suction filtration. The solid was dispersed in 300 mL of CH_2Cl_2 and the mixture was passed through Celite to remove AgI. Ether (300 mL) was added to the filtrate and the precipitate was collected by suction filtration to obtain the title compound 1.18 g in 87% yield as a wine-red microcrystal. No signal could be observed in ^{1}H- and ^{13}C-NMR due to the trace amount of radical species that was generated by the residual water in the deuterated solvent. ^{19}F-NMR (376 MHz, CD_2Cl_2, δ) -78.0. Anal. Calcd. For $C_{40}H_{24}Br_4F_{12}N_4O_8S_4$: C 35.21, H 1.77, N 4.11. Found: C 35.01, H 1.93, N 4.12. Block-shaped wine-red single crystals were grown from the vapor-diffusion method using CH_2Cl_2 and ether under ambient atmosphere. CCDC-2074406.

Synthesis of N^4,N^4,$N^{4'}$,$N^{4'}$–Tetrakis(4–bromophenyl)–[1,1'–biphenyl]–4,4'–diammoniumyl bis(trifluoromethylsulfonyl)imide (TAB-TFSI)

TAB–2TFSI (136 mg, 0.100 mmol) was added in one portion to a solution of TAB (80.4 mg, 0.100 mmol) in CH_2Cl_2 (10 mL) at 0 °C. After stirring the mixture at room temperature for 30 min the solvent was removed under vacuum to obtain the title compound in quantitative yield (216 mg). Anal. Calcd. For $C_{38}H_{24}Br_4F_6N_3O_4S_2$: C 42.09, H 2.23, N 3.88. Found: C 42.05, H 2.49, N 3.88.

Evaluation of EA of the neutral dopants

To systematically discuss the doping capability of TAB–2TFSI against other reported dopants, CV measurement was also conducted using the commercially available compounds (TCNE, TCNQ, and F_4–TCNQ). The EA values of these compounds were estimated using the half-wave reduction potential ($E_{red,1/2}$) (1st half-wave reduction potential for TCNQ and F_4–TCNQ) against the half-wave potential of ferrocene ($E_{fc,\ 1/2}$) which was adjusted to 0 V. As shown in **Figure S1**, the $E_{red,1/2}$ values of TCNE, TCNQ, and F_4–TCNQ were -0.151, -0.193, and 0.177 V, respectively. Assuming $E_{fc,\ 1/2}$ = -5.1 eV against vacuum level, the EA of TCNE, TCNQ, and F_4–TCNQ is 4.95, 4.91, and 5.28 eV.

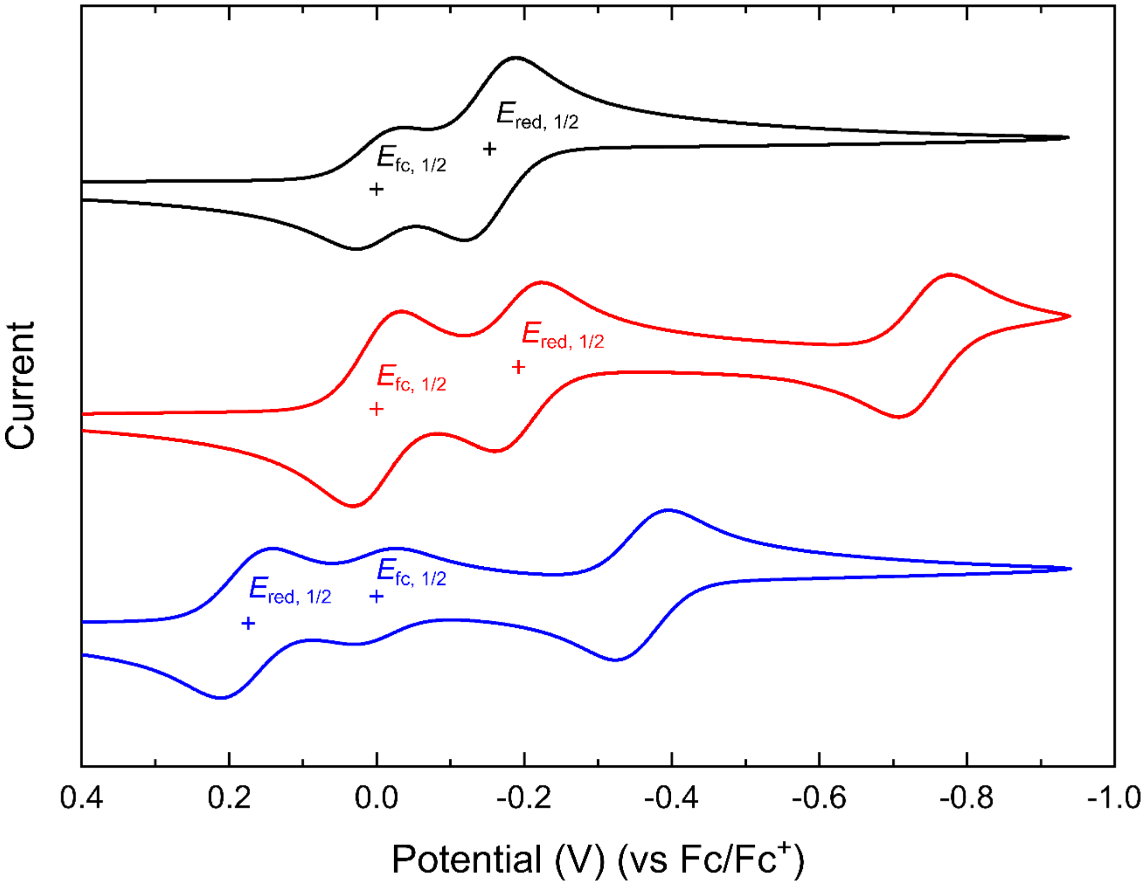

Figure S1. Cyclic voltammograms of TCNE (black line), TCNQ (red line), and F_4–TCNQ (blue line) in acetonitrile with ferrocene. $E_{fc,\ ½}$ was adjusted to 0 V.

We perceive that the conversion of $E_{fc,\ 1/2}$ to the energy level against vacuum level is reported to vary between -4.4 to -5.1 eV and that the choice of this value will strongly affect the discussion regarding the doping capability.[S3] Here, we adopted the value of -5.1 eV because this would provide the EA of the most studied dopant (F_4–TCNQ) to 5.28 eV which is consistent with many other literature. This also allows a fair comparison between the EA of CN_6–CP (larges value of organic dopant reported so far) and the dopant in this work.[S4]

Fitting the absorption spectrum of the aged sample with authentic compounds.

All absorption data of the authentic compounds were normalized with the most intense absorption wavelength and were corrected by multiplying all the data points with the reported molar extinction coefficients at this wavelength (ε_λ) which values are shown in **Figure S2**. The absorption data were simply added by keeping the total abundance ratio to 100% by assuming no other side product was generated and was again normalized to fit the aged sample absorption spectrum.

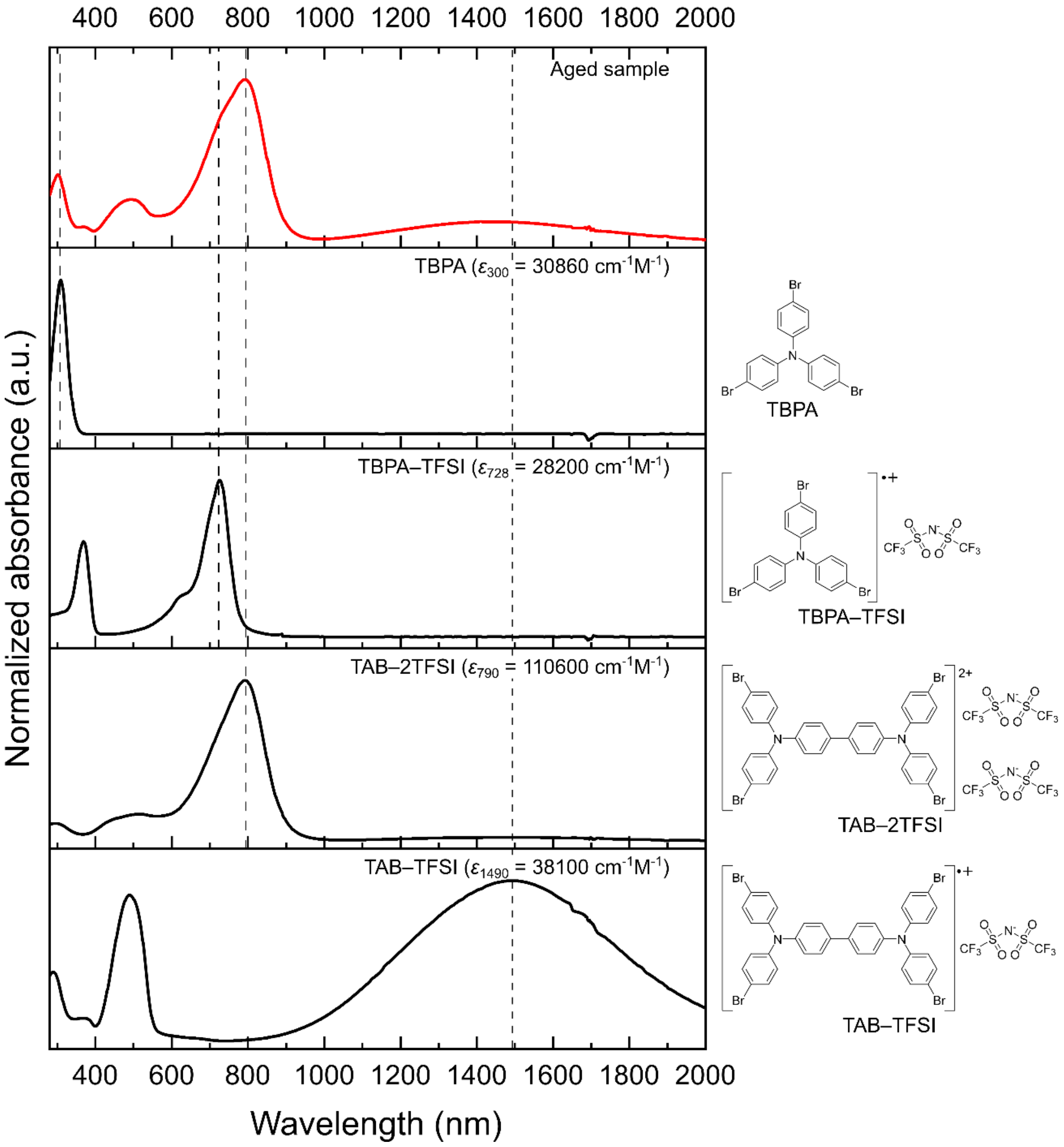

Figure S2. Comparison of UV-vis-NIR spectra between the aged sample and each product of disproportionation prepared separately. Black dashed lines indicate the characteristic absorption wavelengths used for fitting the aged sample spectrum.

Preparation of fresh TBPA–TFSI solution.

The TBPA–TFSI solution was freshly prepared according to a modified procedure of a previous report.[S5] Tris(4-bromophenyl)amine (723 mg, 1.50 mmol, 1.0 mol amt.) and AgTFSI (813 mg, 2.10 mmol, 1.4 mol amt.) were dissolved in ether (50 mL) at room temperature. After complete dissolution of the solid, the system was cooled down to -35 °C using an acetonitrile / dry ice bath and to this solution was added dropwise a solution of iodine (383 mg, 1.50 mmol, 1.0 mol amt.) in ether (15 mL) over 20 min. The mixture was warmed to room temperature and the precipitate (a mixture of TBPA–TFSI and AgI) was isolated by Schlenk filtration technique. The solid was washed thoroughly with dry ether until no coloration of the filtrate was observed. After replacing the bottom flask where the filtrate is stored, the solid was washed with 100 mL of dry CH_2Cl_2 in several portions to completely dissolve TBPA–TFSI and to isolate from AgI. The filtrate was cannula transferred to an oven-dried and Ar-filled Schlenk tube.

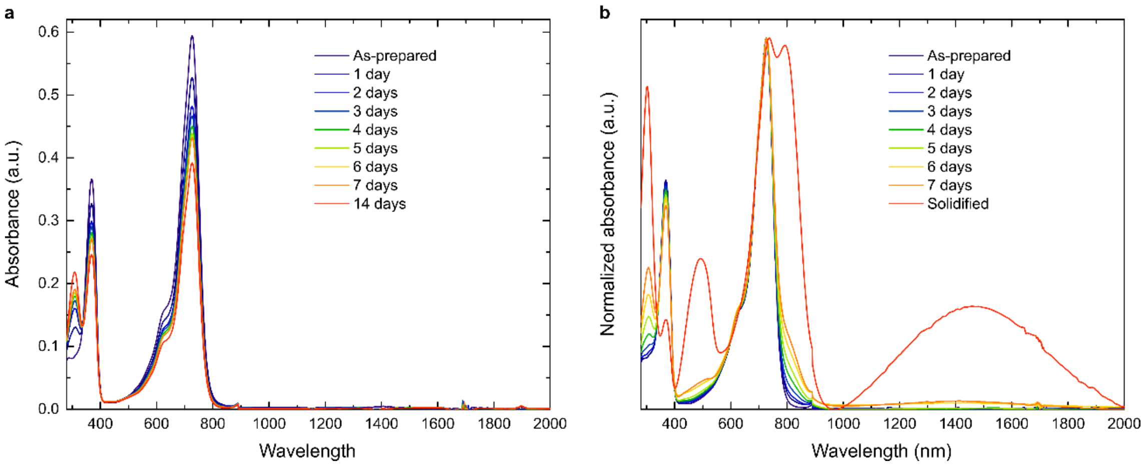

Figure S3. Solution UV-vis-NIR absorption spectra of TBPA–TFSI in CH_2Cl_2 stored at the concentration order of (a) 10^{-5} and (b) 10^{-2} M. The dilute solution was kept in the same cuvette and measured at every interval indicated in the legend while the concentrated solution was kept in an Ar filled sealed Schlenk tube and the solution was diluted 1000 times before every measurement. The "solidified" sample was prepared by removing the solvent under vacuum of the concentrated solution after 7 days of storage and diluted 1000 times. Note that no atmospheric oxygen nor water was in contact during this preparation.

Evaluation of the redox potential of TAB–2TFSI

CV measurement of TAB–2TFSI was carried out to compare the actual redox potential of TAB^{2+} in TAB–2TFSI to the estimated value derived from neutral TAB. The scan direction for each compound is opposite to one another because of the difference in the initial charged state. The calculated doping ability of TAB^{2+} and $TAB^{\bullet+}$ in TAB–2TFSI were −5.67 and −5.57 eV, respectively, which are in good agreement with the estimated values from CV measurement of TAB (−5.73 and −5.53 eV for TAB^{2+} and $TAB^{\bullet+}$, respectively). It is worth noting that CV measurement of TAB–2TFSI and TAB was carried out in acetonitrile and CH_2Cl_2, respectively, because of solubility difference. Hence, the difference in the calculated doping ability derived from the two compounds is mainly from the solvation.[S6]

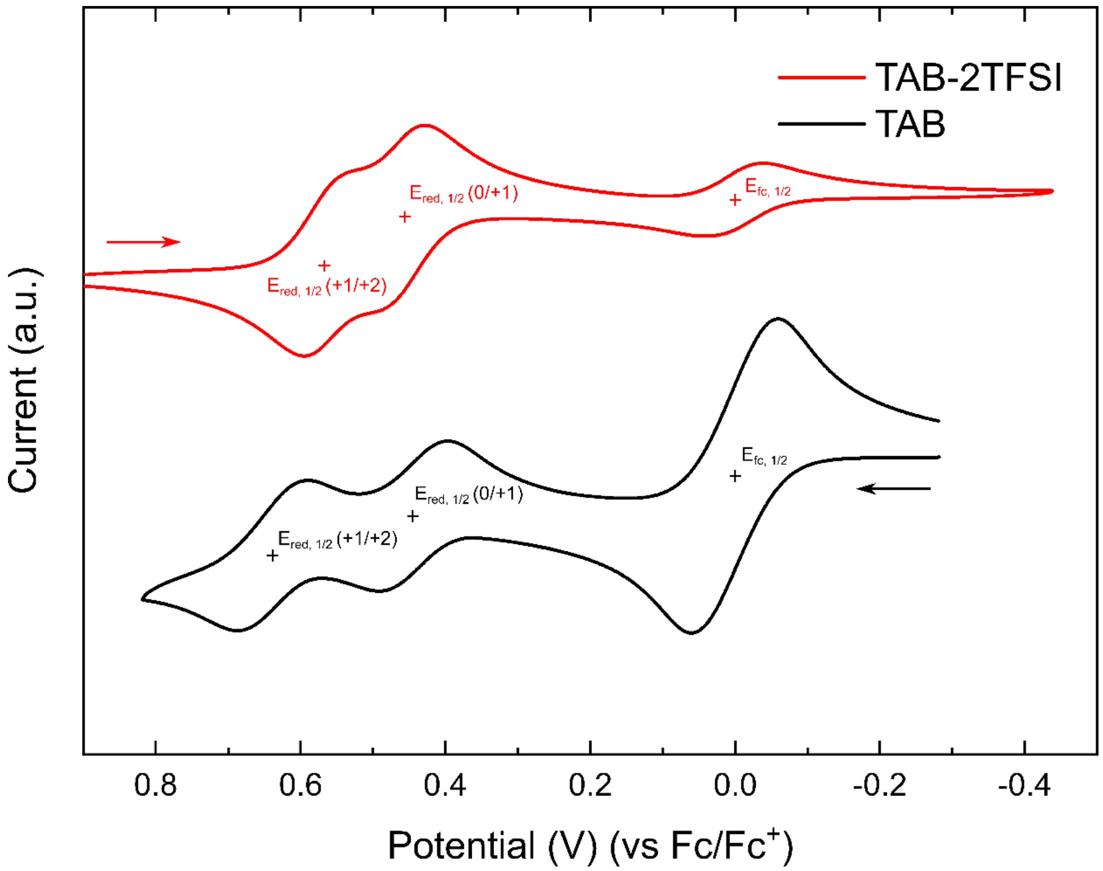

Figure S4. Cyclic voltammograms of TAB (black line) and TAB–2TFSI (red line) in CH_2Cl_2 and acetonitrile with ferrocene. $E_{fc,\,1/2}$ was adjusted to 0 V. The arrows indicate the sweeping direction of the potential.

Evaluation of the aggregated structure in as-prepared TAB–2TFSI

To confirm that the aggregation structure in the as-prepared TAB–2TFSI is similar to that of a single crystal, PXRD measurement was performed using the synthesized sample. Although no crystallization process was carried out in the synthetic procedure, the powder pattern showed a reasonable match as indicated by the dotted line in **Figure S5a**. In particular, a characteristic peak at 11.7° corresponding to (110) diffraction was observed which strongly suggests that the alternate-stack form of TAB^{2+} and $TFSI^{-}$ (**Figure S5b**) exist in the bulk powder.

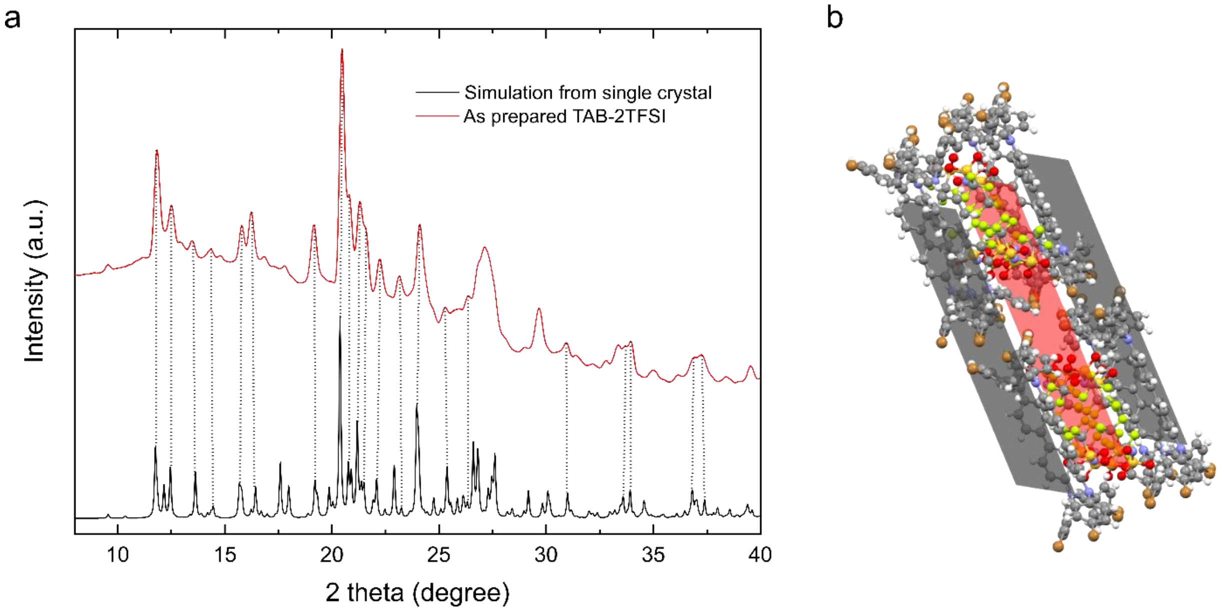

Figure S5. (a) PXRD pattern of as-prepared TAB–2TFSI and simulated pattern from the single crystal analysis data. (b) Diffraction plane of (100).

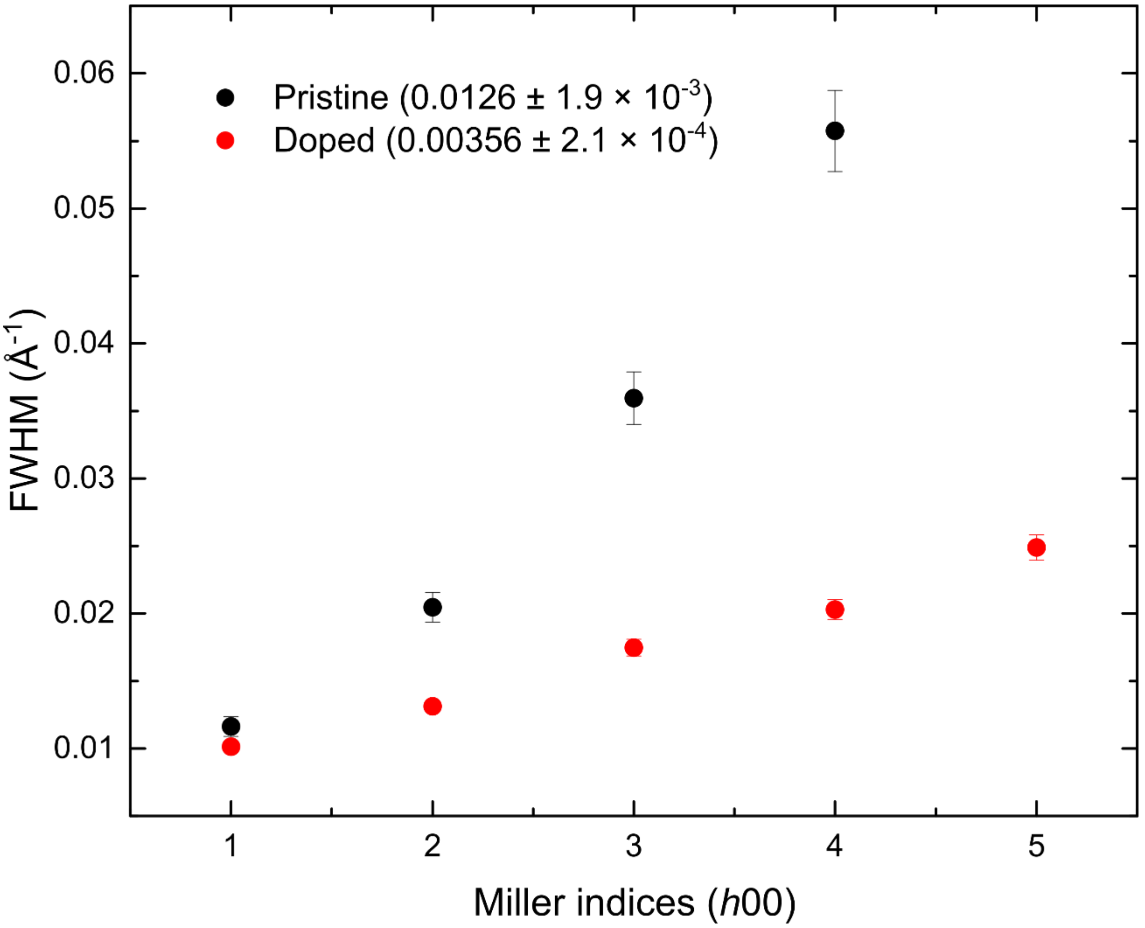

Figure S6. FWHM values of out-of-plane lamella diffraction peaks evaluated by pseudo-Voigt function fitting. The displacements (δFWHM) were determined from the slope of linear fit and are described in the legend.

Fabrication of 2-terminal device, doping of PBTTT–C14 thin film, and conductivity measurement.

The glass substrate (Corning EAGLE XG) was pre-cleaned by sonicating in acetone then isopropanol for 10 min each. Then, chromium (Cr) (3 nm) and gold (Au) (40 nm) were thermally evaporated through a shadow mask under a high vacuum to pattern the electrode with a channel length of 50, 100, 200, 500, and 1000 µm and width of 2000 µm. After evaporation of the Au-Cu electrodes, the device was again subjected to sonication in acetone then isopropanol for 10 min each.

All procedures were carried out in a N_2 filled glovebox unless otherwise noticed. PBTTT–C14 was dissolved in *o*DCB (1 wt%) at 120 °C and deposited on a glass substrate by spin coating (500 rpm for 5 sec then 2000 rpm for 60 sec) while the solution was hot. The thin film was dried and thermally annealed at 180 °C for 30 min and gradually cooled to room temperature. After the film deposition, the polymer was taken out of the glovebox and edged using a yttrium aluminum garnet (YAG) laser to pattern the channel width to 200 µm (**Figure S7a**). Then the film was again taken into the glovebox and doped by immersing in 1.5 mM solution of TAB–2TFSI in acetonitrile at 60 °C for 10 min using a closed vial. The doped film was gently removed from the dopant solution and dried at 60 °C for 10 min.

Current-voltage (I–V) characteristics were measured using KEITHLEY 2400 source meter to evaluate the sheet conductance. After this, the film thickness was measured by directly measuring the edge of the patterned film with atomic force microscopy (AFM) which was determined to be 38$\pm$4 nm (**Figure S7b**). The sheet conductance was determined by taking the slope of the linear fitted I–V curve and the conductivity was obtained by dividing the sheet conductance by the average film thickness.

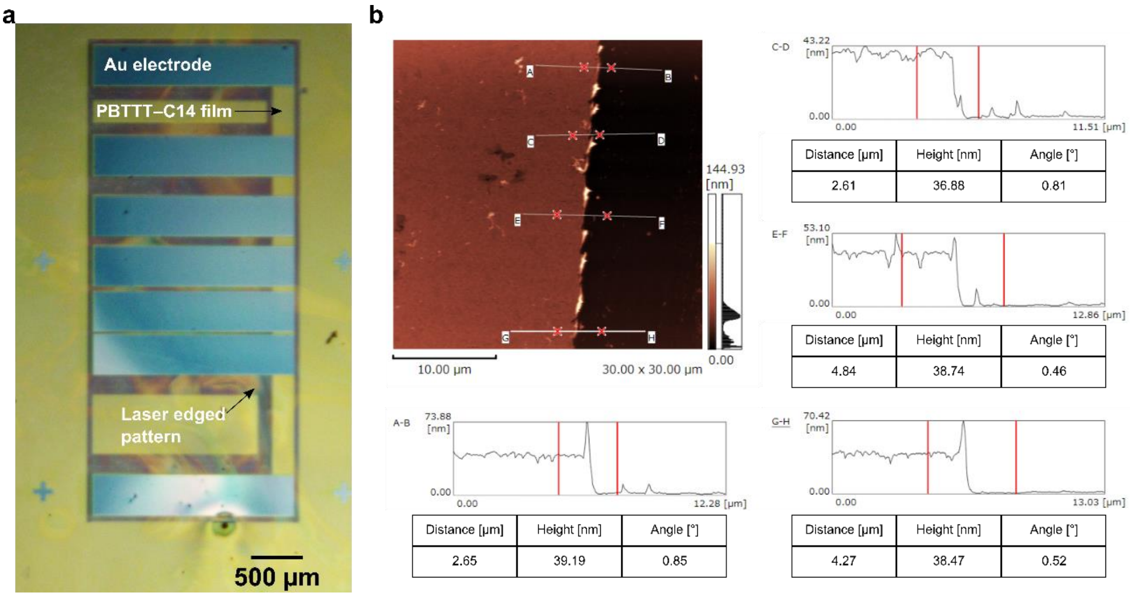

Figure S7. (a) Optical microscope image of a 2-terminal device with laser patterned PBTTT–C14 thin film. (b) AFM height image of doped PBTTT–C14 at the edge of the patterned channel.

Fabrication of 4-terminal device and Hall effect measurement.

Hall effect measurement was performed using a 4-terminal device with Hall bar geometry. The electrodes (Cr (3 nm) and Au (30 nm)) were thermally evaporated on a pre-cleaned EAGLE XG glass substrate. On to this patterned substrate was deposited PBTTT–C14 film via spin coating in the same condition described in the previous section. The deposited film was patterned by YAG laser etching to form a channel length and width of 200 and 40 µm, respectively (**Figure S8a**). After the doping process using TAB–2TFSI, the device was encapsulated with CYTOP 809M (AGC Chemicals).

The 4-point-prove conductivity and Hall effect were measured using a He gas exchanged cryostat with a superconducting magnet (Mercury System, Oxford Instruments) in conjunction with a KEITHLEY 2400 source meter and KEYSIGHT 34465A 6 1/2 digital multimeter. The conductivity and Hall coefficient (R_H) were determined from longitudinal voltage proves (V_1, V_2, and V_3) and transverse probes (V_2 and V_4), respectively. The longitudinal and transverse electromotive forces were both simultaneously monitored while applying a dc current of 1 μA and sweeping the magnetic field from 10 T to −10 T at a rate of 0.2 T/min (**Figure S8b**). The Hall carrier density (n_H) was derived from the inverse Hall coefficient according to $\boldsymbol{n}_H = (e\boldsymbol{R}_H)^{-1}$. The Hall mobility ($\mu_H$) was estimated from the relationship between R_H and conductivity (σ) as $\boldsymbol{\mu}_H = \boldsymbol{R}_H\boldsymbol{\sigma}$.

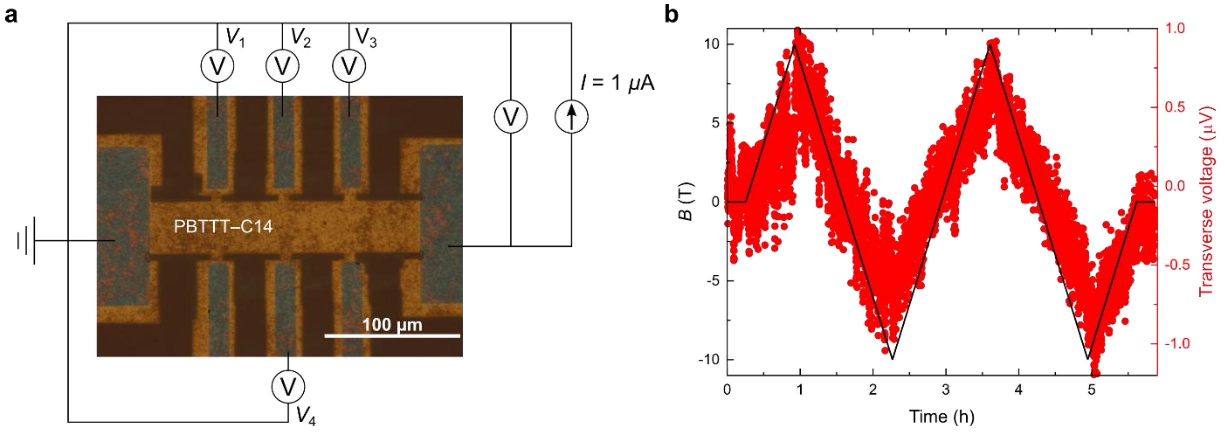

Figure S8. (a) Optical microscope image of a 4-terminal device with Hall bar. (b) Hall voltages were recorded at 250 K and the applied magnetic field B along the out-of-plane direction to the PBTTT–C14 film.

Spin-cast doping of PBTTT–C14 with TAB–2TFSI

The thin film of PBTTT–C14 was prepared in the same procedure described above. To the spinning PBTTT–C14 thin film (3000 rpm), three droplets of TAB–2TFSI solution in acetonitrile (100 mM) were dropped within 5 sec and the spinning was continued at the same rate for another 60 sec. The spin rate was set to this value to ensure no excessive dopant would remain on the device. Finally, the doped film was dried at 60 °C for 10 min.

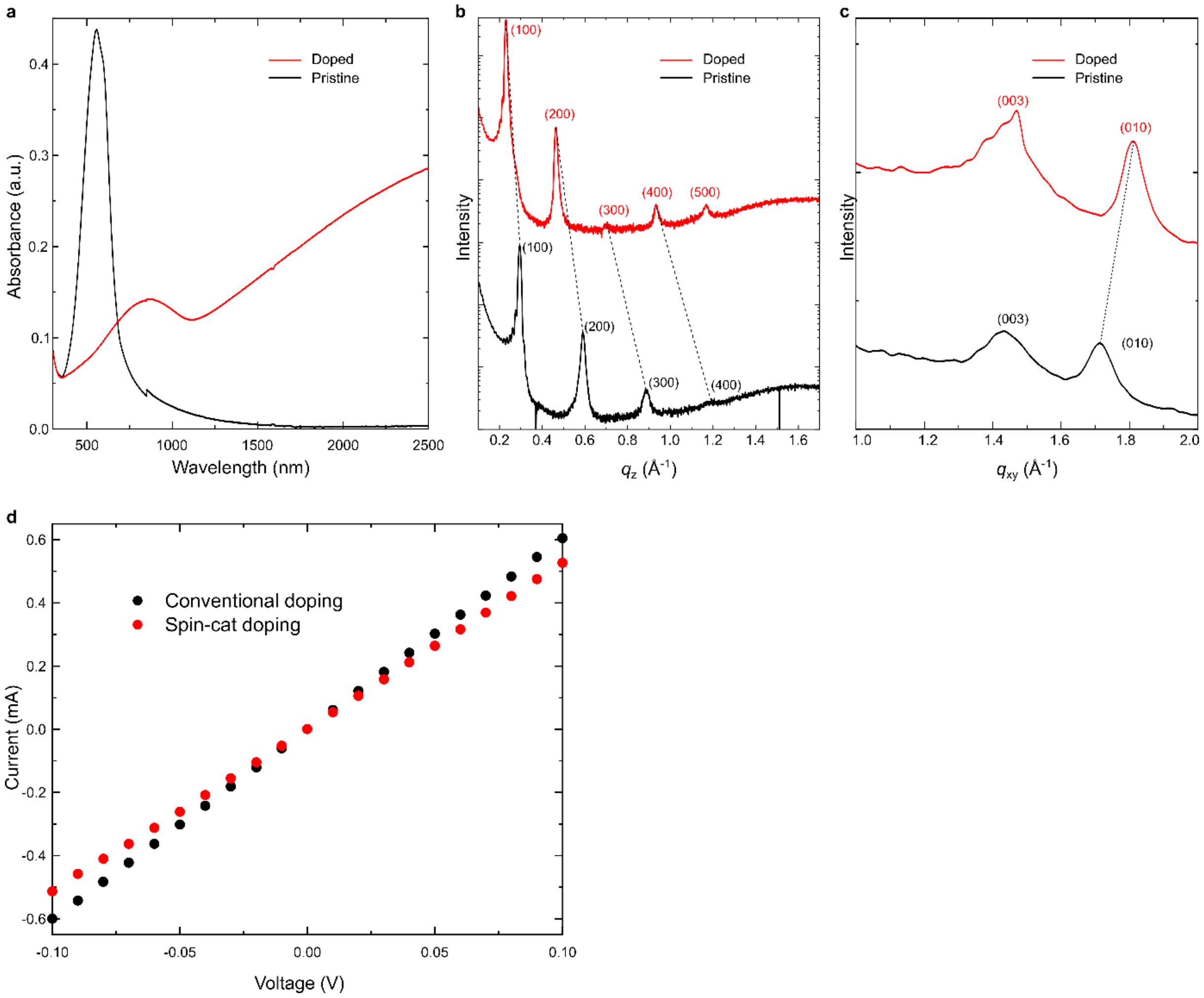

Figure S9. (a) UV-vis-NIR absorption spectra of pristine and spin-cast doped PBTTT–C14 thin film using 100 mM TAB–2TFSI solution. (b) Out-of-plane and (c) in-plane XRD data of the same pristine and doped PBTTT–C14 thin films. (d) Representative I–V plots of the 100 µm channel length 2-terminal devices of doped PBTTT–C14 using conventional and spincast doping procedure.

Verification of proposed doping mechanism of TAB–2TFSI.

The preparation of dopant solution was carried out in a N_2 filled glovebox to avoid any contact with atmospheric moisture. A 1.5 mM acetonitrile solution of TAB–2TFSI was prepared and part of the solution was diluted to a concentration of 3×10^{-5} M (before doping) in a quartz cuvette equipped with a screw cap. The rest of the dopant solution was used for doping PBTTT–C14 film on a glass substrate that was prepared in the same procedure to fabricate 2-terminal and 4-terminal devices. After immersing PBTTT–C14 film at 60 °C for 10 min, the solution was cooled to room temperature and the supernatant was diluted in the same manner described above. After preparing both solution samples, the cuvettes were taken out of the glovebox and were subjected to UV-vis-NIR absorption measurement (**Figure S10a**). Note that the characteristic absorption bands of $TAB^{\bullet+}$ are also present in the dopant solution before doping because the trance amount of water in a highly polar solvent will react with the ionized TAB–2TFSI at a diluted system.

The doped PBTTT–C14 film prepared above was subjected to XPS measurement. To characterize the atomic composition changes before and after the doping process, undoped PBTTT–C14 and TAB–2TFSI powder were also measured (**Figure S10b**). Although XPS is a surface sensitive measurement, the data acquired in this measurement reflects the atomic composition of a few molecular layers from the surface which makes it reasonable to conclude the absence of a TAB unit in the doped film.[S7]

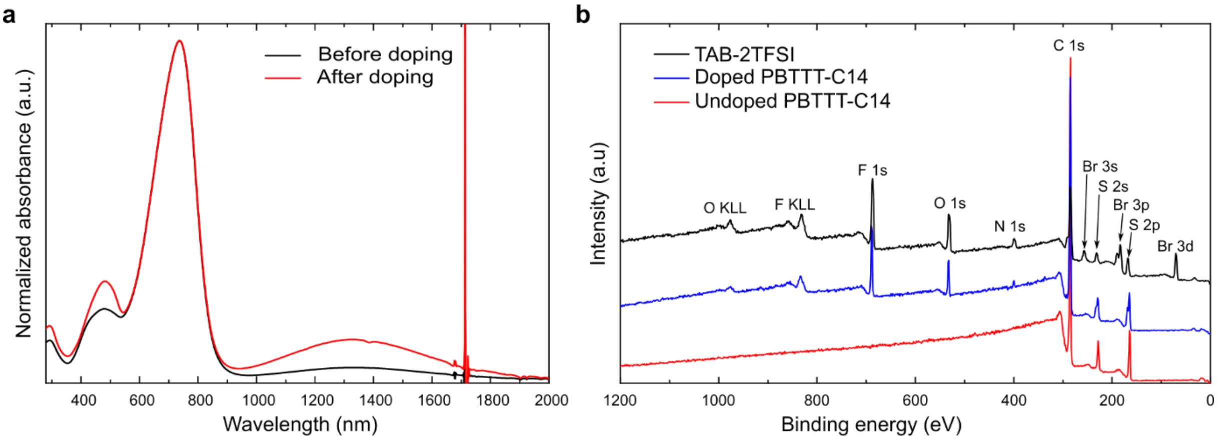

Figure S10. (a) UV-vis-NIR absorption spectrum of TAB–2TFSI solution in acetonitrile before (black) and after (red) doping. (b) XPS wide scan analysis of TAB–2TFSI (black), doped PBTTT–C14 (blue), and undoped PBTTT–C14 (red).

References

[S1] I. McCulloch, M. Heeney, C. Bailey, K. Genevicius, I. MacDonald, M. Shkunov, D. Sparrowe, S. Tierney, R. Wagner, W. Zhang, M. L. Chabinyc, R. J. Kline, M. D. McGehee, M. F. Toney, *Nat. Mater.* **2006**, *5*, 328.

[S2] Q. Huang, G. Evmenenko, P. Dutta, T. J. Marks, *J. Am. Chem. Soc.* **2003**, *125*, 14704.

[S3] C. M. Cardona, W. Li, A. E. Kaifer, D. Stockdale, G. C. Bazan, *Adv. Mater.* **2011**, *23*, 2367.

[S4] Y. Karpov, T. Erdmann, I. Raguzin, M. Al-Hussein, M. Binner, U. Lappan, M. Stamm, K. L. Gerasimov, T. Beryozkina, V. Bakulev, D. V. Anokhin, D. A. Ivanov, F. Günther, S. Gemming, G. Seifert, B. Voit, R. Di Pietro, A. Kiriy, *Adv. Mater.* **2016**, *28*, 6003.

[S5] F. A. Bell, A. Ledwith, D. C. Sherrington, *J. Chem. Soc. C Org.* **1969**, 2719.

[S6] N. G. Connelly, W. E. Geiger, *Chem. Rev.* **1996**, *96*, 877.

[S7] Y. Yamashita, J. Tsurumi, T. Kurosawa, K. Ueji, TsunedaYukina, S. Kohno, H. Kempe, S. Kumagai, T. Okamoto, J. Takeya, S. Watanabe, *Commun. Mater.* **2021**, DOI 10.1038/s43246-021-00148-9.
